# The Role of Oceanic Transform Faults in Seafloor Spreading: A Global Perspective From Seismic Anisotropy

**DOI:** 10.1002/2017JB015176

**Published:** 2018-02-26

**Authors:** Caroline M. Eakin, Catherine A. Rychert, Nicholas Harmon

**Affiliations:** ^1^ Research School of Earth Sciences The Australian National University Canberra ACT Australia; ^2^ Ocean and Earth Science National Oceanography Centre Southampton, University of Southampton Southampton UK

**Keywords:** transform faults, seismic anisotropy, mid‐ocean ridge, seafloor spreading, source‐side splitting

## Abstract

Mantle anisotropy beneath mid‐ocean ridges and oceanic transforms is key to our understanding of seafloor spreading and underlying dynamics of divergent plate boundaries. Observations are sparse, however, given the remoteness of the oceans and the difficulties of seismic instrumentation. To overcome this, we utilize the global distribution of seismicity along transform faults to measure shear wave splitting of over 550 direct S phases recorded at 56 carefully selected seismic stations worldwide. Applying this source‐side splitting technique allows for characterization of the upper mantle seismic anisotropy, and therefore the pattern of mantle flow, directly beneath seismically active transform faults. The majority of the results (60%) return nulls (no splitting), while the non‐null measurements display clear azimuthal dependency. This is best simply explained by anisotropy with a near vertical symmetry axis, consistent with mantle upwelling beneath oceanic transforms as suggested by numerical models. It appears therefore that the long‐term stability of seafloor spreading may be associated with widespread mantle upwelling beneath the transforms creating warm and weak faults that localize strain to the plate boundary.

## Key Points

- Seismic anisotropy beneath oceanic transforms is revealed by global source‐side splitting analysis
- Results are characterized by nulls (60%) and azimuthally dependent splitting
- The pattern found is consistent with widespread mantle upwelling as suggested by numerical models

## 1 Introduction

Most of Earth's crust, both present and in the past, was formed along the global mid‐ocean ridge (MOR) system where two oceanic plates are pulled apart. A fundamental feature of this seafloor spreading is the formation of transform faults of varying length that offset the ridge segments at 90°. This characteristic ridge‐transform geometry is a key component of plate tectonics and governs the creation of new seafloor (Wilson, 1965). Despite the fundamental role of oceanic transform faults, tight constraints on the underlying dynamics have proven challenging due to the inaccessibility of the oceans. Given that transform faults are absent during continental rifting (e.g., the East African Rift) (Pagli et al., 2015), it is unclear why and when transform faults initiate, or how they are maintained over time. The implication is for zones of weakness in the lithosphere upon which strain is localized to ensure long‐term stability of the plate boundary (Gerya, 2012). Elevated levels of aseismic slip, or rather a seismic deficit, also points toward particularly weak faults (Abercrombie & Ekstrom, 2001).

Deformation of the upper mantle is often associated with the development of seismic anisotropy. Plate boundaries, where strain is concentrated, are therefore expected to display strong anisotropic signatures (i.e., directional dependence of seismic velocity). Such anisotropy forms as a result of mantle deformation in the dislocation creep regime (Karato, 2008). This causes a rotation and alignment of individual olivine crystals, of which the upper mantle is mostly composed, producing what is known as a lattice‐preferred orientation (LPO) (Christensen, 1984; Nicolas & Christensen, 1987). By investigating the properties of seismic waves as they pass through the upper mantle, it is therefore possible to deduce the pattern of mantle flow if the relationship between strain geometry and the resulting crystallographic orientation is known. For olivine the type of LPO that develops is dependent on physical and chemical conditions present, such as water content and temperature (Jung et al., 2006; Jung & Karato, 2001; Katayama et al., 2004). Under typical upper mantle conditions A‐, C‐, or E‐type olivine fabrics are expected for which the fast direction, as measured by teleseismic shear waves, is expected to align with the mantle flow direction (Karato et al., 2008; Zhang & Karato, 1995).

Alternatively, seismic anisotropy can also be generated according to a shape‐preferred orientation from layering between two materials of different seismic properties, for example, aligned partial melt (Holtzman et al., 2003). In this case seismic waves travel slowest normal to the layering and fastest in any direction parallel to layering (i.e., transverse isotropy). This type of seismic anisotropy is thought to be prevalent in the shallow crust due to the alignment of stress‐induced cracks and fractures (Crampin, 1994).

Characterizing seismic anisotropy beneath oceanic transforms therefore holds the potential to inform us about the underlying mantle dynamics, distribution of any melt, and the presence of other highly anisotropic minerals such as hydrous phases. Seismic observations over the oceans, particularly of the plate boundaries, are sparse given the difficulty and expense of deploying ocean bottom seismometers (OBS). Some of the earliest studies of seismic anisotropy from the oceanic realm came from seismic refraction surveys (Pn studies), which showed that the uppermost mantle, just below the Moho, was anisotropic with a fast direction parallel to the paleo‐spreading direction (Gaherty et al., 2004; Hess, 1964; Raitt et al., 1969). More broadly, the global pattern of azimuthal anisotropy for the oceanic upper mantle can be described from surface wave observations (e.g., Beghein et al., 2014; Debayle & Ricard, 2013; Schaeffer et al., 2016). Generally these show an alignment of the fast direction with the absolute plate motion in the asthenosphere, and the paleo‐spreading direction in the lithosphere. While surface waves are useful for retrieving information about depth dependency, they tend to average laterally and therefore are not well suited to resolving in detail the plate boundaries.

Arguably, the best method to make detailed point‐based measurements of seismic anisotropy at the plate boundary is with shear wave splitting. When a shear wave enters an anisotropic medium, such as the upper mantle, it is split into two effectively orthogonal polarisations, a phenomenon equivalent to crystallographic birefringence. These two polarisations correspond to a fast (Φ) and a slow orientation and accumulate a delay time (δ*t*) between them due to their difference in seismic wave speed. The magnitude of the delay time depends upon the strength of the anisotropy and the path length through the anisotropic domain. For the same anisotropic domain, the path length, and therefore delay time, may vary for different shear wave phases with different angles of incidence.

**Figure 1:**
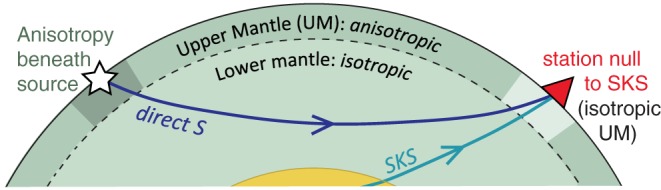
Schematic raypath geometry of the source‐side splitting method. If seismic anisotropy beneath the receiver can be neglected (no splitting of *SKS* phases), and the lower mantle is isotropic, then any splitting of direct *S* phases as shown is attributable to seismic anisotropy in the upper mantle beneath the earthquake source.

Typically, mantle anisotropy beneath a seismic station is derived using teleseismic phases such as *SKS* (Figure 1). These travel through the outer core as a *P* wave, removing splitting accrued on the downward‐leg beneath the earthquake source, and polarizing the upward traveling *S* wave as it emerges from the outer core into the source‐receiver plane (i.e., aligned with the back azimuth). The eventual *SKS* splitting recorded is thus accumulated between the lowermost mantle and the surface beneath the receiver. It is thought that most of the lower mantle is isotropic and that anisotropy primarily resides in the upper mantle, and possibly to a lesser degree in the transition zone (most likely in the vicinity of subducting slabs) (Auer et al., 2014; Chang et al., 2015; French & Romanowicz, 2014; Moulik & Ekstrom, 2014). The lowermost layer of the mantle (D″) is also known to be anisotropic (Kendall & Silver, 1996; Montagner, 1998; Nowacki et al., 2011), but the path length for *SKS* is relatively short compared to the upper mantle.

The continents have been blanketed by such *SKS* splitting measurements (http://splitting.gm.univ-montp2.fr/DB) (Wüstefeld et al., 2009) but the ocean basins and mid ocean ridge ‐ transform fault (MOR‐TF) system remain mostly blank except for a small handful of studies. The MELT (Wolfe & Solomon, 1998) and GLIMPSE (Harmon et al., 2004) experiments traversed a relatively straight and fast spreading ridge segment on the East Pacific Rise (around 114°W, 15°S) and found fast directions subparallel to the spreading direction. Likewise for the Cascadia Initiative, which covered the entire Juan de Fuca plate from ridge to trench, fast directions across the plate and its boundaries were found to align with the large‐scale plate motion (Bodmer et al., 2015; Martin‐Short et al., 2015).

Instead of measuring seismic anisotropy beneath the seismic station, the source‐side technique can be employed to measure anisotropy beneath the earthquake source (Russo & Silver, 1994)(Figure 1). Such a technique has been successfully deployed in numerous subduction settings around the world (Eakin et al., 2016; Eakin & Long, 2013; Foley & Long, 2011; Lynner & Long, 2013, 2014; Russo, 2009; Russo et al., 2010) but has scarcely been applied to other types of plate boundaries (Nowacki et al., 2012). In this technique splitting of teleseismic *S* phases are measured at seismic stations for which the anisotropy beneath the receiver is well known from *SKS* analysis and can be corrected for (or neglected in the case of isotropy). If the lower mantle is mainly isotropic then the remaining splitting on the direct *S* wave should be attributable to anisotropy beneath the earthquake source, hence the term “source‐side.” Event‐station pairs in the 40°–80° epicentral distance range are used for this type of analysis to maintain a relatively steep angle of incidence while avoiding passage through the D″ region.

Recently, Nowacki et al. (2012) conducted the first source‐side splitting study using MOR earthquakes (mostly on the Mid‐Atlantic and East Pacific Ridges) using stations in North America and East Africa. The study focused on ridge events and found that fast directions are subparallel to plate motion away from the spreading center, but closer to the ridge axis fast directions become more variable and splitting times decrease. Azimuthal dependence was identified for two events on the Mid‐Atlantic Ridge, where the fast direction differed between measurements made in North America versus Africa. The limited station distribution, however, restricted further exploration across a broader azimuthal range.

In the present study we conduct a new source‐side (direct *S*) splitting analysis with a sevenfold increase in measurements using a global network of suitable seismic stations. This provides worldwide coverage of the entire MOR‐TF system (subject only to seismicity), sampling the seismically active oceanic transform faults particularly well. Using our global station distribution, the azimuthal dependence of seismic anisotropy beneath transform faults is characterized and modeled on the global scale.

## 2 Data and Methods

### 2.1 Station Selection

For source‐side measurements, as we conduct here, the largest potential source of error is incorrect characterization of the anisotropy beneath the seismic station. For this reason careful station selection is the most important step in the process. Given the restricted distribution of earthquakes in the world, it is usually difficult to record *SKS* arrivals across a wide range of back azimuths, which is critical for conclusively determining the anisotropic structure, for example, single layered or multilayered (Rümpker & Silver, 1998; Silver & Savage, 1994). To best circumvent this complication in this study, we limit ourselves to only null stations. These are stations for which *SKS* splitting analysis has returned an overwhelming majority of nulls (i.e., a clear *SKS* pulse that has not undergone splitting) across a substantial swath of back azimuths. This indicates that the upper mantle beneath the station is effectively isotropic to shear waves with a steep angle of incidence. An initial catalogue of 83 such null stations was compiled from a range of previous studies (Eakin et al., 2015; Foley & Long, 2011; Long, 2010; Lynner & Long, 2013, 2014, 2015; Paul & Eakin, 2017; Walpole et al., 2014). A full list is provided in Table S1 of the supporting information.

#### 2.1.1 Automated *SKS* Analysis

To ensure the reliability of these null stations, we conducted our own *SKS* splitting analysis using an automated approach for speed and efficiency. For each station we selected events of magnitude >6.0 and in the distance range 88°–130° on which to analyze *SKS* splitting. All seismograms were band‐pass filtered between 0.04 and 0.125 Hz. The splitting analysis was performed using the standard SplitLab software package (Wüstefeld et al., 2008). We use the original version of the program SplitLab 1.0.5 in which the error estimation has not been modified according to (Walsh et al., 2013). Typically, the time window around the *SKS* phase is hand‐picked and varied to obtain the best result. In order to speed up the calculation for many thousands of *SKS* events we adapted the code to eliminate the visual inspection routine and instead fixed the time window to ±15 s on either side of the predicted *SKS* arrival time. The signal‐to‐noise ratio using this time window was computed, and events with signal‐to‐noise ratio < 5.0 were discarded. This simple automation technique cannot reproduce the accuracy or detail of visually inspecting each seismogram, particularly for complex anisotropic structures (e.g., Eakin & Long, 2013), but if the stations are indeed characteristically null as previously published, then that should be unquestionably clear with a simplified approach.

Within the SplitLab environment two independent measurement methods are applied over a grid search to determine the predicted fast direction (Φ) and delay time (δt). These two approaches are the minimum energy method (Silver & Chan, 1991) denoted by SC, and the rotation correlation method (Bowman & Ando, 1987) denoted by RC. A comparison of the predicted splitting parameters (Φ and δt) returned by the two different methods provides a simple diagnostic tool for classifying splits and nulls as outlined by Wüstefeld & Bokelmann (2007). If anisotropy is present, then the two methods should predict similar splitting parameters. For a null, however, the RC method tends toward a delay time of zero and a systematic deviation of Φ_RC_ by 45°. This results in a delay time ratio (δ*t*_RC_/δ*t*_SC_) close to unity for a split and close to zero for a null. Additionally, the angular difference (ΔΦ) between predicted fast directions (Φ_SC_ − Φ_RC_) tends to zero for a split and toward 45° for a null. An example of this classification system for station CBKS is shown in Figure S1. A station was dropped from our list if the percentage of “good” or “fair” nulls was less than 80% of the total (splits and nulls). The average total number of measurements per station was 205.

**Figure 2:**
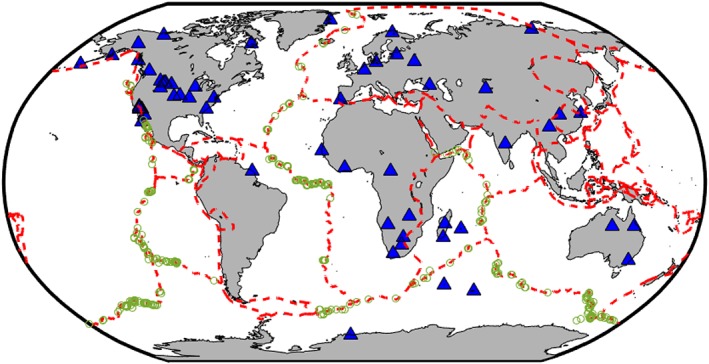
Map of seismic stations (blue triangles) and oceanic transform events (green circles) used in this study. A full list is provided in the supporting information. Plate boundaries (dashed red line) from Bird (2003).

We also assessed the results as a function of back azimuth to check for consistency (Figure S2). We require that nulls are not just found at one particular back azimuth but instead fall over a wide swath (minimum 50°). This ensures that the upper mantle structure below the station is apparently isotropic to all such phases and not just the result of back azimuth alignment with a fast or slow direction, which would have a clearly identifiable 90° periodicity. Following this inspection 24 stations were cut from our list, leaving us with 56 null stations with robust apparent mantle isotropy below (Table S1 and Figure 2). The stations that were not redeemed robust may have implications for previous source‐side studies.

#### 2.1.2 Station Misalignment

**Figure 3:**
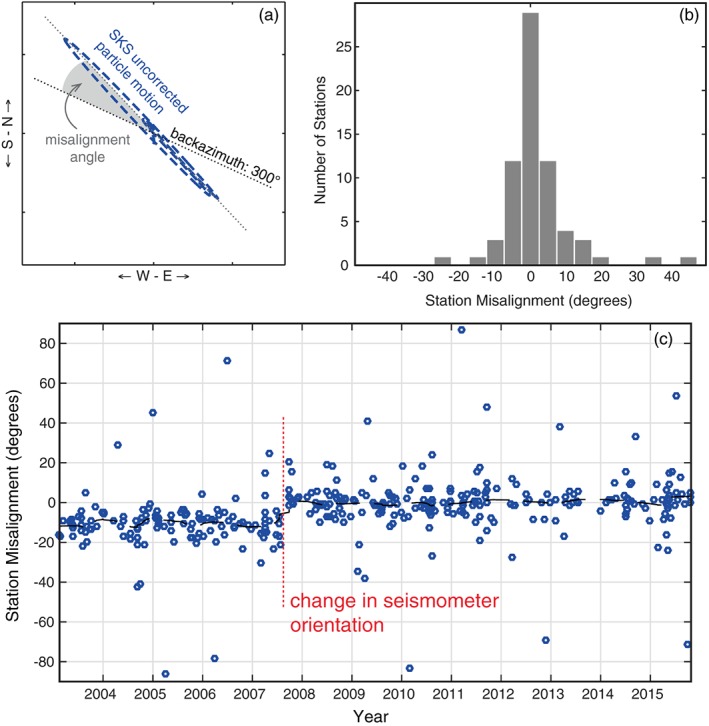
Estimating seismic station orientation from *SKS* initial polarization. (a) Difference between the uncorrected *SKS* particle motion (north versus east components) and the source‐receiver back azimuth reveals the misalignment angle. (b) Histogram of the average misalignment estimated from all *SKS* events at each station. Most stations appear correctly orientated (misalignment = 0°), but misalignment errors of ±15° are not unusual. (c) Example of misalignment estimates as a function of time for station CBN. The black dashed line is the moving average. Orientation of the seismometer appears to change in mid‐2007. Corrections to station orientations are therefore applied as a function of time. Misalignments for other stations are provided in the supporting information.

When making accurate shear wave splitting measurements, another potentially significant source of error is the orientation of the seismic station (Tian et al., 2011). Previous studies have shown that the reported azimuth of the horizontal components can be off by 10° or more due to the difficulty of orientating a seismometer in the field (e.g., Ekstrom & Busby, 2008). As it so happens analysis of *SKS* polarization provides an alternative method for calculating the station orientation (Vidale, 1986). Due to the polarization effect of traveling as a *P* wave in the outer core, *SKS* phases are initially aligned to the back azimuth. By observing the horizontal particle motion of *SKS* phases and comparing to the known source‐receiver back azimuth, the station misalignment can be determined (Figure 3a).

We included this procedure as part of our automated *SKS* analysis. This was achieved by measuring the angle of the first eigenvector (longest axis) of the *SKS* particle motion from the north and east components. If the *SKS* phase is not split (i.e., null), as most events were, then the initial particle motion is linear and the orientation of the first eigenvector is well defined. Even in the case of splitting, the initial particle would be elliptical, with the long axis of the ellipse aligned with the back azimuth.

From this type of analysis, it was found that for the majority of stations, the average misalignment angle is close to zero (Figure 3b); that is, the correct station orientation is known. Upon closer inspection, however, it was noticed that for some stations estimates of the misalignment angle vary substantially. When these stations are plotted as a function of time (Figure 3c), a step can usually be seen where the misalignment suddenly changes. It is likely that the seismometer was moved at this point in time during instrument servicing. In most cases the misalignment improves suggesting that a known problem was being fixed. A table of all the stations used in this study and their misalignment values are provided in Table S1 for future reference. Using these values, a time‐dependent correction was applied to the stations before subsequent source‐side splitting analyses were made.

### 2.2 Source‐Side Analysis

Following the steps outlined previously, we are left with 56 null stations distributed around the world (Figure 2) that are reliable, and in the correct orientation, ready for source‐side splitting analysis to be undertaken. Using these stations, we search for suitable earthquakes of magnitude 5.5 and above in the epicentral distance range 40°–80° from each station. This returns 1,337 individual events spanning the entire global MOR‐TF system, many of which are recorded across multiple stations and locations (each event recorded by 3.2 stations on average). For the purposes of this study we focus on 995 of the events (74% of the data set) with strike‐slip source mechanisms (Ekström et al., 2012) associated with oceanic transform faults (Figure 2). Results relating to the remainder of the events can be found in the supporting information.

Using these stations, we measured shear‐wave splitting on the direct *S* phase in a manner similar to that described earlier for *SKS* analysis (section 2.1.1). The same two methods, SC and RC, are applied through SplitLab, and the waveforms are analyzed in the same frequency band (0.04–0.125 Hz) to negate any complications associated with frequency dependence. For the SC method, the splitting parameters are estimated by minimizing the energy on the component orthogonal to the initial polarization direction (i.e., the polarization of the shear wave before it encounters anisotropy). Unlike for *SKS* phases in which the initial polarization is known, for direct *S* phases, the initial polarization requires calculation. This we estimate from the long axis of the ellipse in the uncorrected particle motion, which preserves the initial polarization direction when splitting times are small relative to the dominant period of the *S* phase (Eakin & Long, 2013). A comparison between this method and others for estimating the initial polarization is shown in Figure S3 (Marson‐Pidgeon & Savage, 2004; Wolfe & Silver, 1998). While both the RC and SC methods are used for comparison and quality control, henceforth, the reported splitting parameters are from the RC method as it is independent of the initial polarization.

Previously, for *SKS* analysis, the process was automated for speed, but for the source‐side measurements, we wish to be as careful as possible so we revert to visual inspection of every seismogram for accuracy. This allows for several additional quality measures to be implemented for source‐side splitting measurements (Figure S4). Namely, a characteristic shear wave pulse must be clearly visible above the noise on both horizontal components (rotated with respect to the initial polarization), both with a similar shape but separated by a small time delay. The component normal to the initial polarization should be flat (i.e., energy minimal) following correction for splitting, and the corrected particle motion linearized in the initial polarization direction. The uncorrected particle motion should be elliptical, not circular, to ensure that the small delay time approximation holds for calculating the initial polarization. The error regions for the estimated splitting parameters were required to be circular and relatively small (0.5 s in δ*t* and 22.5° in Φ) at the 95% confidence level. A similar degree of agreement, that is, within this standard error range, was required between the separate RC and SC estimates. In the case of a null source‐side result, a clear shear wave pulse should be visible on the component parallel to the initial polarization but not on the perpendicular component, producing linear uncorrected particle motion.

Finally, anisotropy beneath the source is sensed by downgoing rays, but splitting is measured at the station from upgoing rays. Due to this difference in the frame of reference (upgoing versus downgoing), the fast directions need to be reflected about the great circle path (i.e., azimuth) to project back to the true orientation beneath the source.

## 3 Results

**Figure 4:**
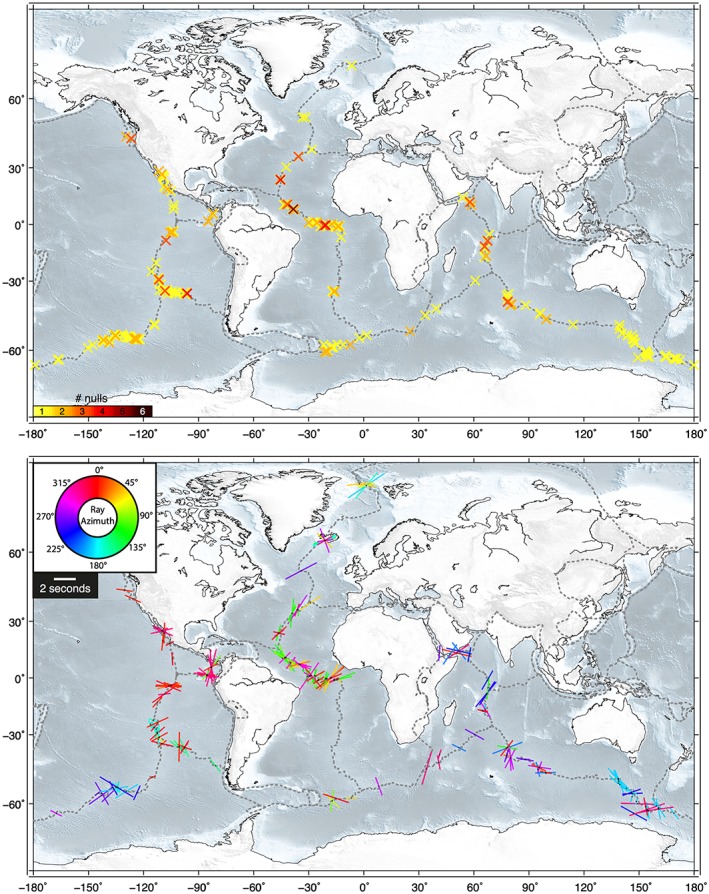
Source‐side splitting results from global oceanic transform earthquakes. Results are plotted at the source location. For the upper map, the number of null measurements (per event) is shown by a colored cross according to the color scale given. Nonnull measurements, that is, splits (colored bars), are shown on the lower map. The orientation of the bar represents the measured fast direction, and its length is scaled by the delay time found. An example for 2 s is given in the legend. The bars are colored based on the azimuth of the raypath (azimuth from the source pointing toward receiver) according to the color wheel provided.

Our analysis yielded 556 source‐side measurements from 367 transform events (Table S2), which we plot at their event locations in Figure 4. The majority of the results (60%, 332/556) were null observations indicating that these shear waves did not undergo any splitting. The remaining 40% (224 out of 556) did show splitting. Of these split results, the mean delay time is 1.7 s (mean error ±0.4 s), which is fairly substantial. However, their fast directions (orientation of colored bars in Figure 4b) are variable, showing no clear trend, even at individual locations. Most stations (49 out of 56) recorded both null and split observations, indicating that the predominance of null results is not a receiver‐side effect. The characteristics are similar when ridge events are also included in the analysis (Table S2 and Figure S5).

**Figure 5:**
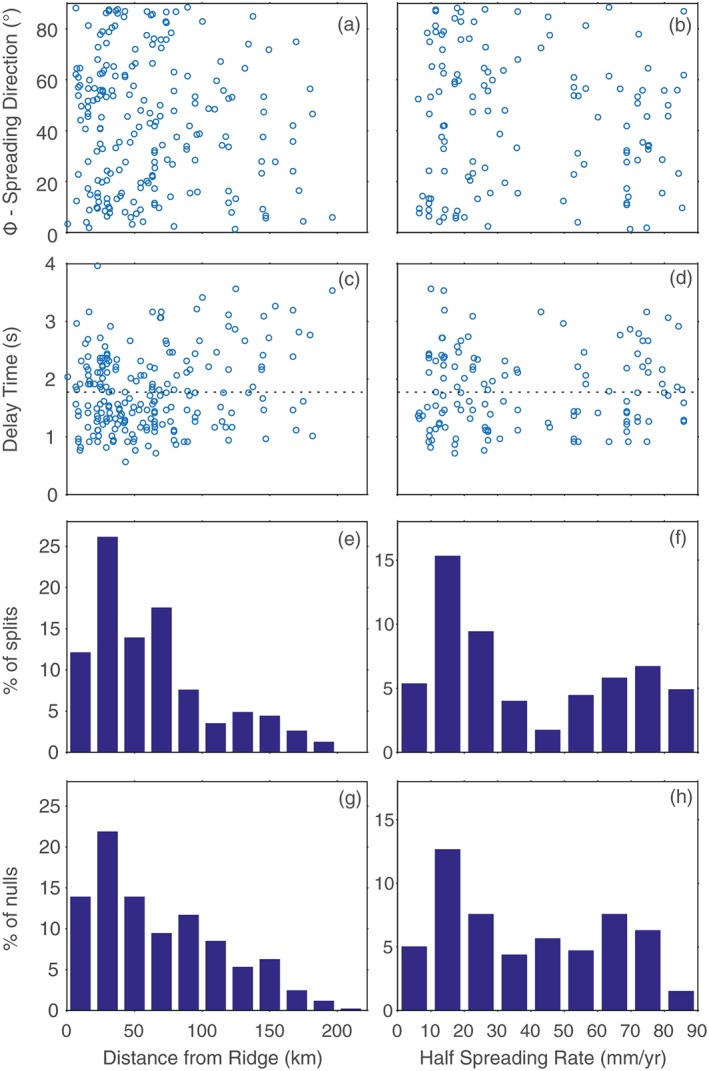
Distribution of results for transform fault events as a function of distance from the closest spreading ridge (left column) and spreading rate (right column) from (Müller et al., 2008). The left column is equivalent to distance along the transform, with the distance to the nearest ridge segment from (Bird, 2003) chosen. (a and b) The absolute angular difference between the measured fast direction (Φ) and the local orientation of seafloor spreading from NUVEL1A (DeMets et al., 1994). (c and d) The distribution of measured delay times. The mean value (1.8 s) is plotted as a dotted line. (e and f) The percentage of splits (as a fraction of the total number of splits) that occurs within a given bin. (g and h) The same for nulls. The pattern as a function of distance and spreading rate is similar for both split and null measurements.

When compared against the spreading direction, the distribution of fast orientations from transform events appears uniform, with no clear preference for spreading parallel or spreading normal, that is, ridge‐parallel orientations (Figure 5a). This holds true even as a function of distance from the spreading center. The probability of splits versus nulls is also unaffected by the distance from the spreading ridge (Figures 5e and 5g); instead, both are tied to the available distribution of seismicity and are equally likely to occur at any set distance. There is little change in average observed delay times with distance from the ridge axis either (Figure 5c). The same is true when making comparisons with the spreading rate (Figures 5b, 5d, 5f, and 5h). Delay times and the preponderance of splits versus nulls are similar for both fast spreading ridges and slow spreading ridges. There therefore appears to be little difference in the general anisotropic characteristics beneath fast versus slow transforms.

### 3.1 Comparison With *SKS* Studies

**Figure 6:**
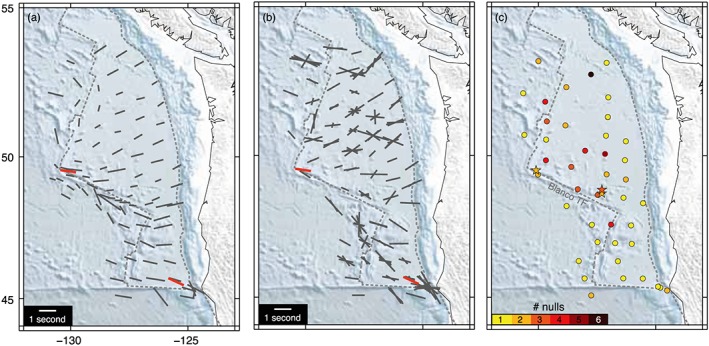
Comparison of source‐side splitting results and *SKS* splitting from the Cascadia Initiative. Only offshore stations west of the trench are shown. (a) Stacked splitting results from Bodmer et al. (2015) plotted as black bars. Our two source‐side splits from the region are plotted in orange, corresponding to a NNE azimuth (see color scale from Figure 4). (b) The same as for (a) but comparing with individual split measurements of Martin‐Short et al. (2015). (c) Number of individual null measurements made at each station by Martin‐Short et al. (2015) are represented by circles colored according to the scale below. Numbers of null source‐side measurements for three events (stars) near the Blanco Transform Fault are also shown.

Where we have *SKS* splitting measurements from broadband OBS deployments, we can compare with our results. Only a handful of such experiments near oceanic transforms have ever been conducted, which in part motivated this study. Results from the most extensive deployment to date, the Cascadia Initiative, covering the entire Gorda‐Juan de Fuca plate from ridge to trench, are shown in Figure 6 (Bodmer et al., 2015; Martin‐Short et al., 2015). In this region, our two new source‐side splitting measurements (in orange) return very similar splitting characteristics, in terms of both fast direction and delay time (the orientation and size of the bar), compared to the nearby *SKS* results (Figures 6a and 6b). This confirms that the source‐side method we have employed is correctly capturing the anisotropic properties beneath the earthquake source.

We also record seven null measurements in the region, near the Blanco Transform Fault (Figure 6c). Bodmer et al. (2015) did not measure nulls as part of their study, so we are only able to compare with the null results from Martin‐Short et al. (2015). For the three stations closest to the Blanco Transform Fault, and to our source‐side events (stars), multiple null results are recorded at each station (orange to red circles). Across the deployment as a whole, most stations record zero or one null *SKS* measurement (no circle or yellow circle). The relative number of splits at the three closest stations also appears reduced (Figure 6b), with two stations having only 1 splitting measurement, and the third having three. Near to the transform fault, the general characteristics of our source‐side results therefore appear to be in agreement with the individual *SKS* results with a greater tendency for nulls and a similarity in the more limited splitting. For the Bodmer et al. (2015) study only the stacked splitting results are available, but we do note that the number of events used to build the stack is on average less for the “Blanco” region (3.4 events per station; 32 stations total), compared to the rest of the data set (5.5 events on average across 84 stations).

The only other location on an oceanic transform fault where there are SKS measurements with which to compare is in southern Iceland. Given the anomalous tectonic setting, with likely plume interactions at play, we discuss these results in Figure S6 (Stefánsson et al., 1993; Xue & Allen, 2005). We note, however, that again the splitting characteristics are consistent between source‐side and *SKS* splitting methods.

### 3.2 Azimuthal Dependence

**Figure 7:**
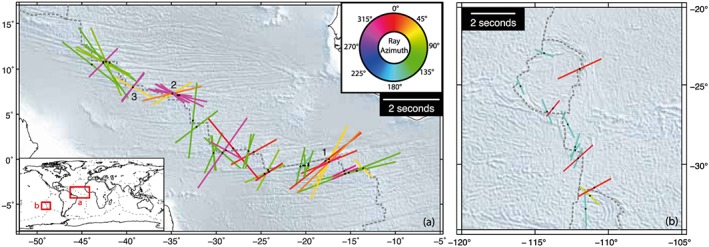
Regional case examples from the (a) central Mid‐Atlantic Ridge and (b) East Pacific Rise illustrating the azimuthal dependence of source‐side splitting results. The coloring and positioning of the bars is the same as Figure 4. Numbers (1, 2, and 3) refer to individual event clusters discussed in the text. Inset global map provides the locations of both Figures (red boxes).

Given our global network of null stations (Figure 2), we were often able to measure source‐side splitting from the same source location across multiple stations in different parts of the world. In 25 different locations we have four or more source‐side splitting measurements for the same event or event cluster (closely spaced events separated by less than 1°) (Figure S7). This allowed us to consider and discover the presence of azimuthal dependency in our results. We find that the variability in fast directions seen in the splitting results (Figure 4b) appears related to the azimuth of the raypath between the event and the station. When we plot the splitting results and color‐code them by azimuth (Figures 4b and 6), we find that in a given location similar azimuths (i.e., similar colors) tend to produce similar splitting characteristics. Conversely, when measurements are made across different azimuths (i.e., the bars are different colors), the splitting characteristics will tend to differ also. For example, along the East Pacific Rise at 30°S, 110°W, there is a cluster of splitting measurements in both red and cyan (Figure 7b). Those in pink‐reddish colors have northerly azimuths (recorded in North America) and tend to display NE‐SW fast directions, in opposing orientation to those in cyan, which have southerly azimuths (recorded in Antarctica).

The complexities and intricacies of the azimuthal dependency become even more apparent if we focus on the central Mid‐Atlantic Ridge region where there is an abundance of splitting (Figure 7a). Again, similar colors and similar azimuths tend to produce similar fast directions and delay times, while different azimuths shown by different colors give different results. At the eastern corner of the Romanche Transform (0°N, 18°W, cluster #1 on Figure 7a) a gradual increase in the ray azimuth from 0 to 50° represented by pink‐red to orange‐yellow produces a clear rotation of the observed fast direction from ENE‐WSW to NNE‐SSW. It does not seem, however, that a universal azimuthal relationship exists as the pattern can change rapidly from one ridge segment to the next. For example, at the Doldrums Transforms (8°N, 35°W, cluster #2), the eastern cluster of splits displays SE‐NW magenta fast directions (azimuth ~300°) and NE‐SW orange fast directions (azimuth ~45°). Meanwhile, less than 500 km to the west (cluster #3), the pattern is reversed, with magenta fast directions now orientated NE‐SW and orange fast directions SE‐NW. We note that for both Doldrums events, each splitting measurement is made at a different station, and that the consistency in results for similar azimuths (e.g., for stations in North America with an azimuth of ~300°) is not due to the same receiver, but seen by multiple receivers, separated by considerable distance (Figure 2), but of similar azimuth. This is demonstrated by the stereo‐plots in Figure S7.

## 4 Discussion

Our source‐side splitting analysis has revealed a complex pattern of anisotropy beneath the global system of transform faults. It suggests the oceanic paradigm of azimuthal anisotropy aligned with seafloor spreading (Maggi et al., 2006; Montagner & Tanimoto, 1991; Nishimura & Forsyth, 1988) does not hold true within the immediate vicinity of the plate boundary. This is not wholly unsurprising given that transform faults mark the dividing line between two opposing plate motions, and therefore two opposing mantle flow directions that must connect at the plate boundary.

We can, however, outline several key characteristics of our data set that any credible interpretation should be able to explain. First, and most importantly, the majority (60%) of our results are nulls. Second, the 60:40 ratio between split and null measurements is consistent across all spreading rates and does not vary with distance along the transform (Figure 5). Third, the 40% splits display clear azimuthal dependence. Given that we do not expect much change in the focal mechanisms between similarly located events, the initial polarization should remain similar also. This means that the azimuthal dependence seen cannot be attributed to multiple layers of anisotropy, as would typically be the case for *SKS* receiver‐side splitting.

Bearing the above in mind, and our predominance of nulls, we first discuss the common ways in which null measurements can be widely generated. First, a lack of coherent seismic anisotropy (i.e., mantle isotropy) could exist beneath transform faults. This could be due to strong heterogeneity (Eakin et al., 2015; Rümpker & Silver, 1998; Saltzer et al., 2000) or irregular mantle flow. While an isotropic upper mantle would arguably satisfy the majority of the results (60% nulls), deformation of the mantle is expected to be concentrated near plate boundaries and so widespread isotropy beneath transform faults where a strong gradient in mantle deformation is required seems unlikely.

Second, nulls can be expected when the incoming polarization of the shear wave is aligned with the fast or slow direction (in the plane orthogonal to the raypath) (Savage, 1999), or for similar reasons when two anisotropic layers exist with a 90° difference in Φ between the layers (Eakin et al., 2015; Silver & Savage, 1994). The source polarization of event clusters should, however, be similar, given that focal mechanism does not change along a single transform fault. This could potentially explain the nulls, given that a common relationship exists between the fault geometry, focal mechanism, and source polarization. If this were indeed occurring, however, then we would not expect to see nulls and splits for the same event or event cluster. This is clearly not the case. As is seen in Figure 4, both nulls and splits are found together in most earthquake locations. Such a mechanism is therefore not possible to explain the 60% nulls on a global scale, but limited individual cases may exist.

**Figure 8:**
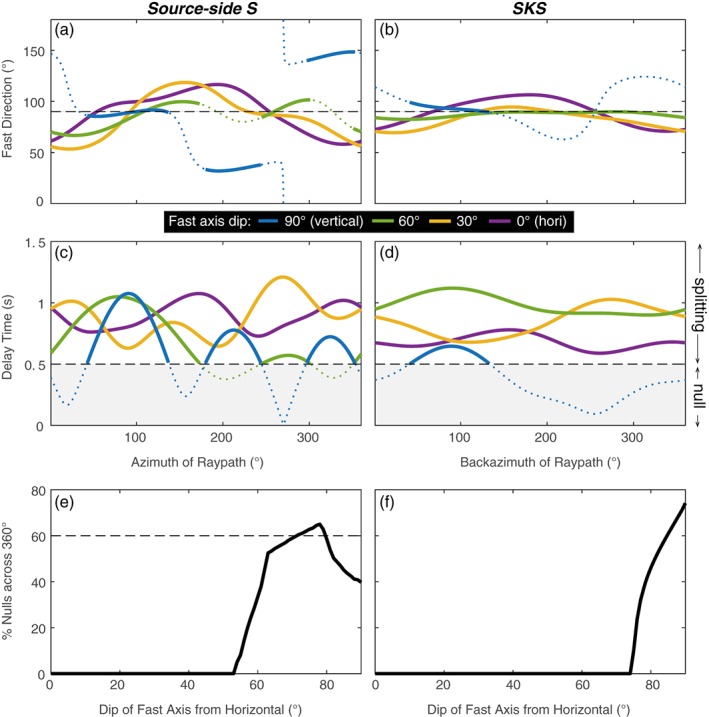
Predicted splitting parameters (a–d) for a 100 km thick layer of the upper mantle with a LPO of olivine (A‐type: Karato, 2008). The left column shows predicted values across all azimuths for typical source‐side ray geometries (average inclination = 24°), while on the right the same is shown for *SKS* geometries, which have steeper raypaths (inclination = 10°). The different colors, as shown in legend, represent the dip angle between the modeled fast *a* axis of olivine (as in Figure S9) and the horizontal. The dashed black line (a and b) represents the dip direction. For (c and d), the grey shaded region and black dashed line signify the 0.5 second splitting cut‐off. Predicted delay times smaller than this amount (dotted colored lines) are below the limit of detectability for teleseismic shear waves and would equate to a null measurement. Azimuth of the raypath as plotted on the *x* axis is the same quantity as that represented by the color wheel in Figure 4. Strong azimuthal dependence, and a mixture of splitting and nulls, appears when the fast axis of anisotropy approaches vertical. For (e and f), the percentage of predicted nulls across all azimuths is shown as a function of fast axis dip. This is equivalent to how often the predicted delay time falls below the 0.5 second cut‐off in (c and d) for a given dip angle. In (e) the percentage of nulls found by our source‐side splitting data set (60%) is given by the dashed black line.

Alternatively, the upper mantle could display a form of anisotropy with a near vertical symmetry axis, in which case velocities in the horizontal plane are similar in any direction but comparably faster or slower in the vertical direction (i.e., radial anisotropy). For a teleseismic shear wave with a steep angle of incidence (e.g., *SKS*), such a vertical symmetry axis would cause weak to no splitting, as the rays would travel close to the symmetry axis. For our moderately inclined raypaths (inclination ~25°), this could generate a mix of nulls and splitting, as well as azimuthally varying splitting parameters, depending on the dip angle between the symmetry axis and the raypath. This is demonstrated in Figure 8 for a classic A‐type olivine LPO example. The elastic constants for the LPO fabric (Table S3) are derived from experiments on olivine aggregates under conditions typical of the upper mantle (see page 407 of (Karato, 2008)). Such A‐type is characterized by a fast symmetry axis whereby the fast *a* axes of the individual olivine crystals tend to align with the direction of shear (i.e., mantle flow direction) (Zhang & Karato, 1995). There are several other known types of olivine LPO fabrics but A‐type is the most commonly found in natural samples, particularly for ridge peridotites (Michibayashi et al., 2016).

Taking our olivine LPO example, we therefore explore different dip angles of the fast symmetry axis by rotating the elastic tensor using the Matlab and Seismic Anisotropy Toolkit (Walker & Wookey, 2012) (Figure S7). For the complete 360° azimuthal range we can then calculate the predicted splitting parameters for typical source‐side and *SKS* ray inclinations by solving the Christoffel equation. This predicts the polarization of the fast quasi‐*S* wave as wells as the strength of the *S* wave anisotropy. By assuming a 100 km thick layer of anisotropy we can then generate an estimate of the splitting delay time. A predicted delay time of less than 0.5 s is designated as a null measurement as this falls below the limit of detectability for teleseismic *S* wave frequencies, as evidenced by the minimum delay time recorded in our data set (Figures 5c and 5d).

From Figures 8c–8f it is seen that, in general, the tendency for null results increases (i.e., across a wider range of azimuths) as the dip angle of the fast symmetry axis steepens. For typical source‐side ray inclinations, nulls are predicted for 60% of azimuths (as we find in our data set) when the fast axis is dipping around 75° from the horizontal. In addition, as the dip angle approaches vertical, the azimuthal variability in the splitting parameters (Φ and δt) increases (Figures 8a–8d) and is more pronounced for source‐side than for *SKS*. Even when the fast axis is vertical, it is still possible to generate some *SKS* splitting, which may explain, for example, why *SKS* splitting is found along the Blanco Transform Fault (Figure 6) when mantle upwelling has been otherwise inferred in the same location (Byrnes et al., 2017). Conversely, when the fast axis is horizontal or shallowly dipping, no null measurements are expected for either *SKS* or source‐side. A steeply dipping or near‐vertical symmetry axis of anisotropy would therefore be able to explain all the main characteristics of our data set, namely, a 60:40 ratio of nulls to splits with azimuthally dependent splitting. For A‐type olivine fabric this would imply vertical mantle flow. Other mechanisms for producing anisotropy, such as shape‐preferred orientations could also achieve a similar effect. If, for example, we consider a model for aligned melt inclusions (Figures S8 and S9 and Table S4 (Blackman & Kendall, 1997; Holtzman & Kendall, 2010; Murase & McBirney, 1973; Tandon & Weng, 1984)) that is characterized by a slow symmetry axis, then either a vertical or intermediate (~45–60°) dip of this symmetry axis could also generate 60% source‐side nulls and azimuthal variability in Φ and δt (Figure S8).

Given that there appears to be no clear relationship between the fast direction and the orientation of seafloor spreading (Figure 5a), we can only constrain the probable dip angle of an anisotropic symmetry axis, but not its dip direction (at least in the global sense). This may be possible on an event by event basis, but only a handful of azimuths are ever sampled for a given event (Figure S7) meaning that any such attempt at modeling at present would be highly nonunique. Additionally, comparing event clusters 2 and 3 in Figure 6a, it appears that even for the same seafloor spreading and transform geometry the azimuthal pattern can change rapidly between one transform segment to the next.

**Figure 9:**
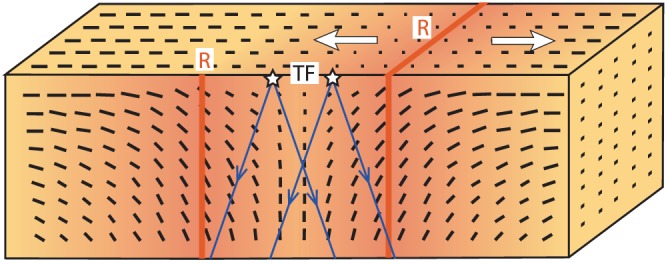
Schematic interpretation of upper mantle deformation beneath transform faults (TF) based on modeling of our source‐side splitting results. A cross section along the transform fault is shown, perpendicular to the adjoining ridge (R) segments. The black bars representing mantle flow are modified from the numerical modeling studies of Morgan & Forsyth (1988) and Behn et al. (2007), which suggest mantle upwelling directly beneath transform faults. This is in agreement with our global catalogue of splitting results measured from direct *S* rays with an average inclination of ~25° (blue arrows).

From the first order view of seafloor spreading it might be expected that oceanic transform faults should display horizontal shear in the mantle parallel to the transform given the strike‐slip motion along the fault. Numerical models of simplified ridge‐transform systems (Behn et al., 2007; Morgan & Forsyth, 1988; Shen & Forsyth, 1992; Weatherley & Katz, 2010), however, tend to display mantle upwelling at asthenospheric depths directly beneath the transform (Figure 9). In these models, vertical velocities tend to zero away from the plate boundary, are highest along the ridge axis, but display intermediate values beneath the transform fault. This vertical flow pattern appears to be induced to accommodate opposing plate motions on either side of the plate boundary, with the horizontal differential motion gradually distributed over a zone surrounding the fault. The horizontal velocities themselves are typically small directly beneath the transform. It is worth noting that this pattern is consistent for both passive (e.g., Morgan & Forsyth, 1988) and dynamic (i.e., buoyancy driven) systems (e.g., Sparks et al., 1993), although the more complex buoyancy‐driven flows may contribute to a lack of dependence on the spreading orientation. Additionally, the predicted anisotropy from both types of systems is consistent with near vertical alignment near the ridge (Blackman & Kendall, 2002), but a direct comparison along a transform fault has yet to be done. It is known, however, that geodynamical models that incorporate a realistic viscoplastic rheology further enhance mantle upwelling beneath the oceanic transforms (Behn et al., 2007; Roland et al., 2010).

The pattern of our results, our predominance of nulls, and our suggestion of an olivine (A‐type or similar) LPO fabric with a steep fast axis are therefore consistent with the predicted mantle flow field from geodynamical models. At present, it is difficult to explicitly state, however, what the lateral extent of mantle upwelling beneath transforms would need to be due to the depth dependency of the shear wave Fresnel zone (width of sensitivity). Future modeling work to directly compare 3D geodynamical models of mantle flow with the observed splitting along oceanic transforms should, however, hopefully provide an answer.

Alternatively, a steep or intermediate slow axis of symmetry from aligned melt pockets would also be consistent with our data set (Figure S8). It is unclear, however, whether such a uniform distribution of melt could persist globally beneath the ridge‐transform system, particularly across a range of spreading rates and transform fault length scales. On the other hand, deformation of the upper mantle and the development of LPO should be fairly ubiquitous (e.g., Park & Levin, 2002). This is particularly true in the vicinity of plate boundaries where strain between tectonic plates tends to be concentrated. It therefore seems that on the whole the anisotropic signature seen beneath transform faults is more likely to be due to coherent mantle flow rather than well‐organized partial melt. Additionally, recent evidence from Byrnes et al. (2017) has been found to support widespread mantle upwelling along the full length of the Blanco Transform Fault (Figure 6), as inferred from low shear wave velocities tomographically imaged in the upper mantle, and in agreement with the predictions from geodynamical models.

MORs (spreading centers) are the primary locales for mantle upwelling and the associated production of new seafloor. The possibility of transform faults, however, as secondary narrow zones of upwelling has the potential to explain several puzzling observations. For example, upwelling is likely to warm the fault, lowering the viscosity, and thus helping to maintain a zone of weakness (i.e., shear localization) that stabilizes the plate boundary (Behn et al., 2007; Bercovici, 2003). A warmer thermal profile is also able to better explain the depth distribution of seismicity on oceanic transform faults (Behn et al., 2007; Roland et al., 2010) and may account for increasing evidence for sporadic magmatism along some transform faults, particularly fast slipping faults such as the Siqueiros on the East Pacific Rise that have been mapped at high resolution (Gregg et al., 2007). This phenomenon is also sometimes referred to as “leaky transforms” (Menard & Atwater, 1969). Upwelling of the mantle below transform faults would promote melting and may aid off‐axis melt migration (Hebert & Montesi, 2011). This may encourage the development of intra‐transform spreading centers, particularly during changes in plate motion (Fornari et al., 1989; Lonsdale, 1989). Further evidence for upwelling and melting can be found from negative residual mantle Bouguer gravity anomalies, from which partial crustal accretion along some oceanic transform faults has been suggested (Gregg et al., 2007). This prompted Bai & Montési (2015) to demonstrate the ability to extract melt along fast spreading transforms when the melt permeability barrier reaches a shallow depth.

It therefore appears that there is sufficient evidence to support mantle upwelling, at least to some extent, beneath transform faults globally, consistent with a near‐vertical fast‐axis of mantle anisotropy. As seen in Figure 9, the mantle flow pattern and resulting anisotropic structure is likely more complicated than we can model for any given raypath at present. Even for one single transform fault, although the predicted flow pattern is generally steeply inclined, the dip varies in direction and inclination, becoming shallower closer to the surface and toward the ridge segments. The evolution of anisotropy and its geometry will therefore vary depending on the exact location of the earthquake along the fault and the azimuth of the raypath. In order to fully account for this in modeling regional specific mantle flow scenarios would be needed for each fault. Other factors such as combined melting and LPO effects (Holtzman et al., 2003), as well as mantle serpentinization from seawater percolation into transform faults (Francis, 1981), could present likely added complications to the overall anisotropy signature.

## 5 Conclusion

We have presented a new suite of source‐side splitting observations that detail seismic anisotropy beneath transform faults around the world. The pattern suggests an anisotropic geometry with a subvertical axis of symmetry, consistent with geodynamic models of mantle upwelling beneath oceanic transforms (Behn et al., 2007) and evidence of sporadic magmatism (Bai & Montési, 2015; Gregg et al., 2007). Such a scenario implies warming, and therefore weakening of transform faults, enhancing shear localization and long‐term stability of divergent plate boundaries. Knock‐on effects for heat flow, melt distribution, and global crustal production may also occur.

In order to fully understand the deformational processes along such plate boundaries, modeling studies with realistic geometries are needed as well as targeted OBS studies to better illuminate the subsurface structure. Recent seismic deployments, such as the PILAB experiment over the equatorial Mid‐Atlantic Ridge (http://www.pilabsoton.wordpress.com), are expected to deliver further insights in the near future. Overall, it is hoped that our new global data set will provide much needed constraints for future investigations of MOR‐TF dynamics.

## Supporting information



Supporting Information S1

Data Set S1

Data Set S2
