# Supplementary material for: The Role of Oceanic Transform Faults in Seafloor Spreading: A Global Perspective From Seismic Anisotropy
Source: J Geophys Res Solid Earth. 2018 Feb 26;123(2):1736–51. doi: 10.1002/2017JB015176 (PMC5993317; doi:10.1002/2017JB015176)
Supplement: Supplementary file 1 — Supporting Information S1 [file JGRB-123-1736-s001.pdf]

Journal of Geophysical Research: Solid Earth

Supporting Information for

The Role of Oceanic Transform Faults in Seafloor Spreading: A Global Perspective from Seismic Anisotropy

Caroline M. Eakin[1,2*], Catherine A. Rychert[2], Nicholas Harmon[2]

[1] *Research School of Earth Sciences, The Australian National University, Canberra, Australia*

[2] *Ocean and Earth Science, University of Southampton, National Oceanography Centre Southampton, Southampton, UK*

* Corresponding Author (caroline.eakin@anu.edu.au)

Contents of this file

Figures S1 to S9
Tables S3 to S4

Additional Supporting Information (Files uploaded separately)

Captions for Tables S1 to S2

Introduction

Nine supplementary figures and two tables are provided, as well as captions for two data tables uploaded separately. These show examples of the station selection procedure (Fig. S1-S2) and shear wave splitting methodology (Fig. S3-S4) as described in the main text. We provide a global map in Figure S5 that includes results from all intra-oceanic events, not just from transforms. Individual localized examples of azimuthal dependence are given in Figures S6-S7. Lastly, examples and discussion of potential anisotropic models are presented (Fig. S8-S9, Tables S3-S4).

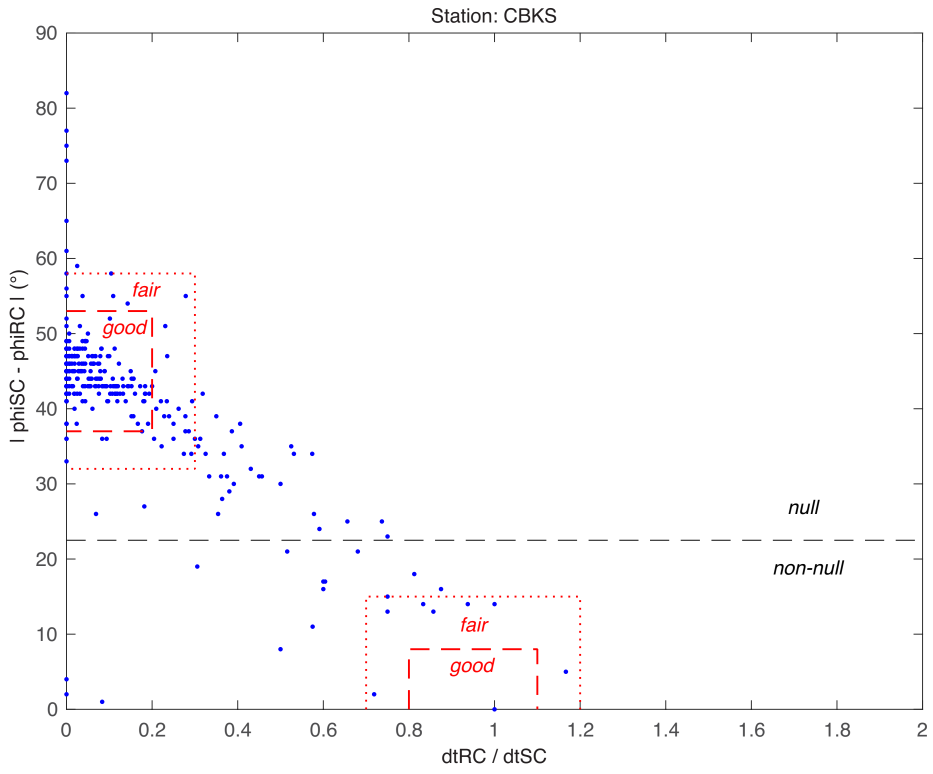

Figure S1. Identification of null versus non-null *SKS* splitting measurements based on comparison predicted splitting parameters Φ (phi) and δt between the [*Silver and Chan*, 1991] energy minimisation (SC) method and the rotation correlation (RC) method [*Bowman and Ando*, 1987]. Classification system is based on the work of [*Wüstefeld and Bokelmann*, 2007]. Measurements which fall within the limits of the dotted red box are considered of 'fair' quality and those within the dashed red box of 'good' quality. An example for station CBKS is shown indicating an overwhelmingly null station (>98% nulls). This procedure is repeated for automated *SKS* splitting at all stations. Only those stations with 80% or more nulls (fair or good quality) are kept.

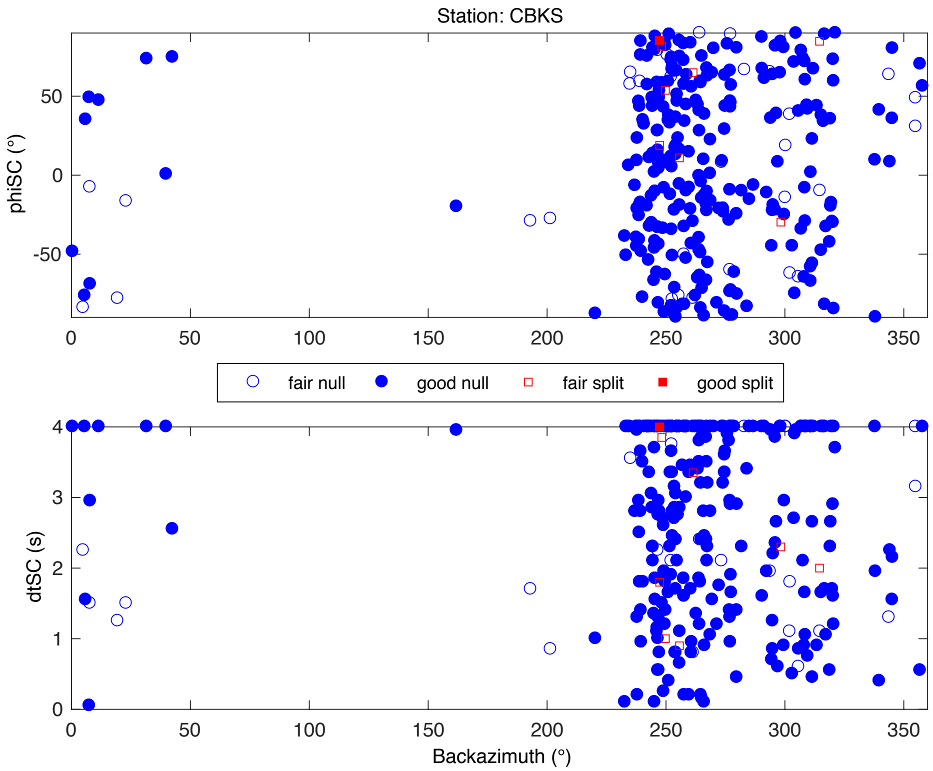

Figure S2. An example of *SKS* back-azimuth distribution check for station CBKS. No clear back-azimuth trend (e.g. 90° periodicity) is seen. A density of null measurements (blue circles) is also present across a wide swath (~230°-360°). Only a few splits (red circles) are inter-mixed with the nulls suggesting that they are likely due to spurious correlated noise.

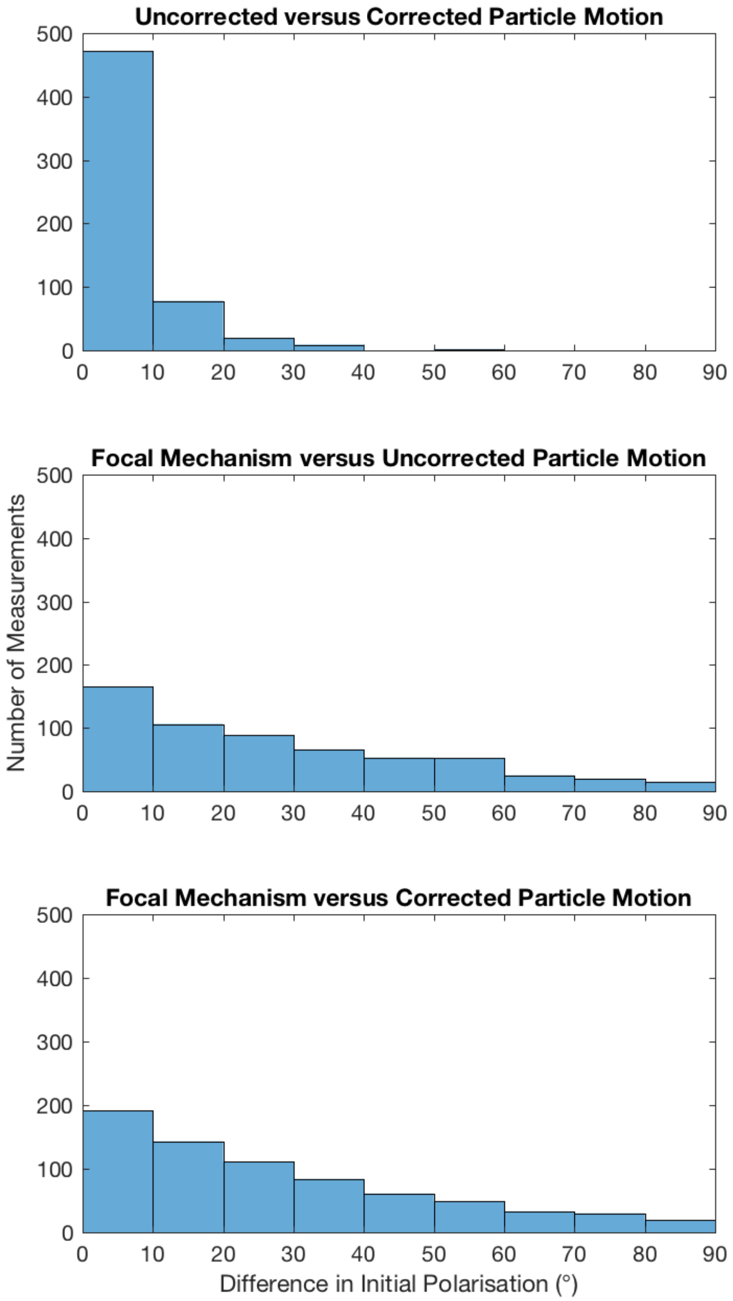

Figure S3. Comparison of three different methods for estimating the initial source polarization of direct *S* phases as applied to our dataset. The three methods shown are (i) from the corrected (i.e. linearized) particle motion after correcting for the effect of shear-wave splitting, (ii) from the uncorrected (i.e. elliptical) particle motion, and (iii) calculated from the global CMT focal mechanism [*Ekström et al.*, 2012] according to [*Wolfe and Silver*, 1998; *Marson-Pidgeon and Savage*, 2004]. The

uncorrected and corrected particle motions give very similar estimates (<20°), while the focal mechanism estimate is in less agreement but still skewed towards zero. This may be due to uncertainty in the focal mechanism itself, which may be relatively poorly constrained for intra-oceanic settings.

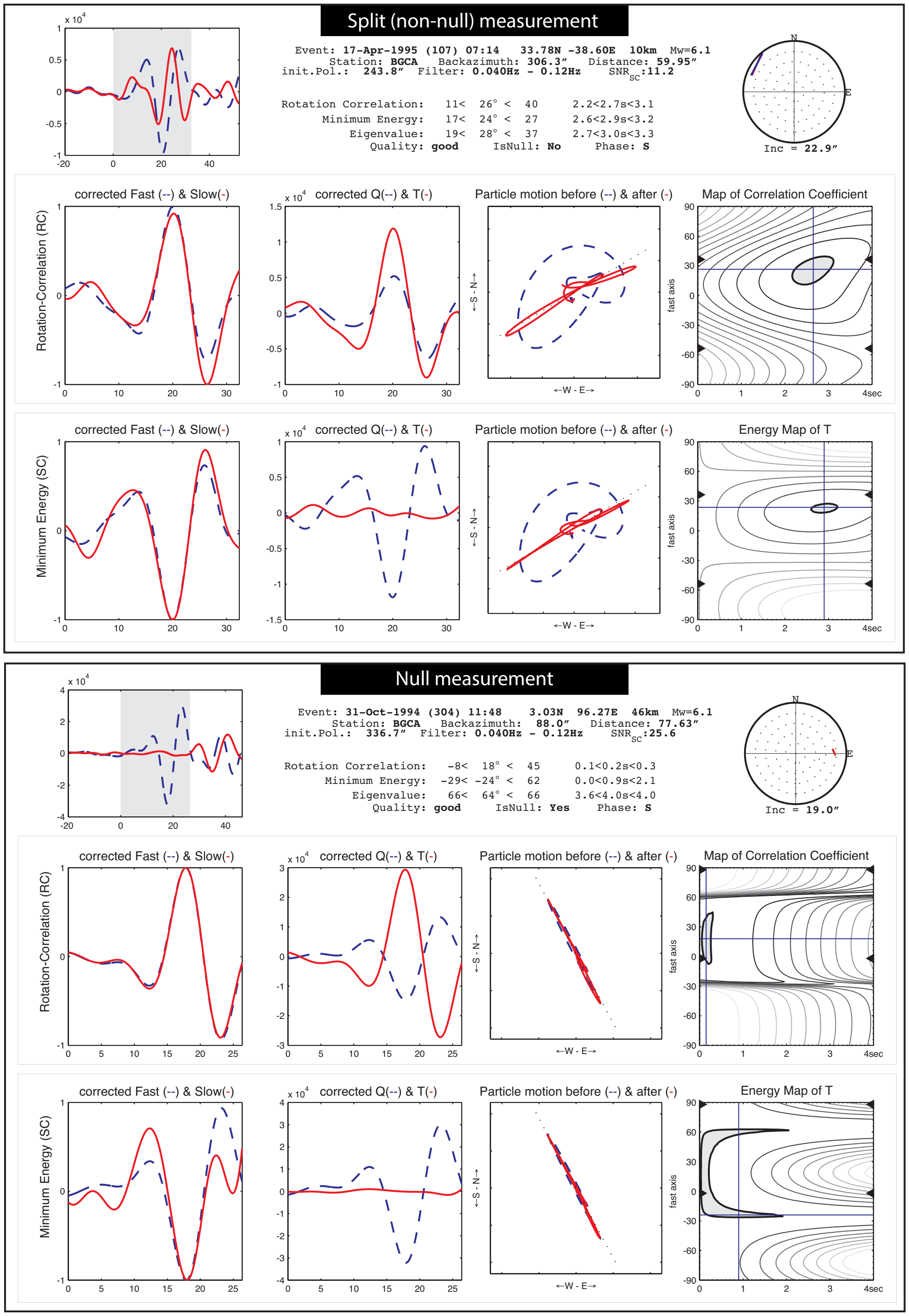

Figure S4. Example of the diagnostic plots generated by the SplitLab user interface for a split and null measurement [*Wüstefeld et al.*, 2008] for station BGCA. Example shown is for splitting of direct *S* (source-side) phases. In this case Q (radial) and T (transverse) refer to components parallel to and perpendicular to the initial polarisation direction, as estimated from the long axis of the uncorrected particle motion (dotted line in 3rd column).

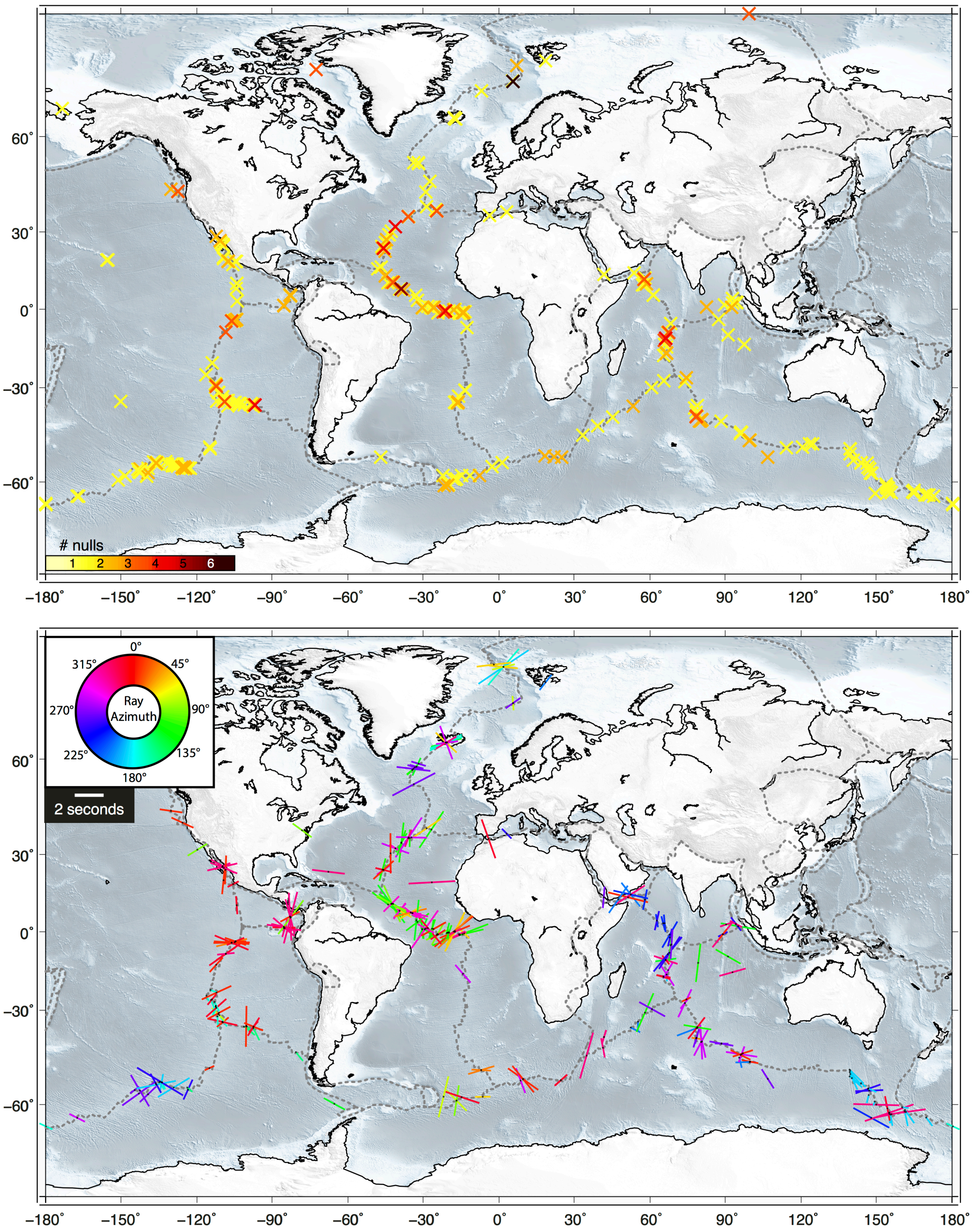

Figure S5. Same as Figure 4 but for all events and results listed in Tables S1-S2. These include events on the mid-ocean ridges with normal source mechanisms, as well as anomalous intra-oceanic events (e.g. from volcanic islands). Results from ridge events are also dominated by nulls and display similar azimuthal dependence of splitting.

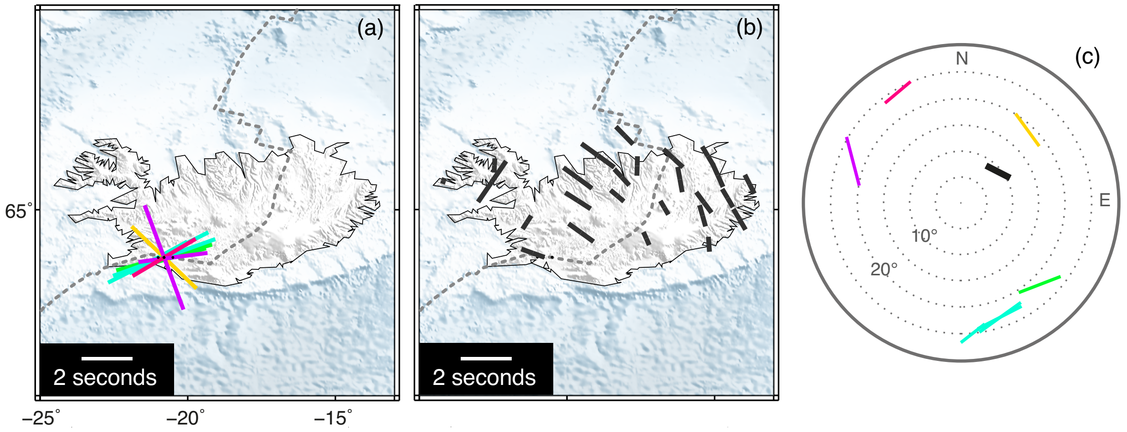

Figure S6. Regional comparison of azimuthally dependent source-side splitting (a) in the Southern Iceland Seismic Zone with *SKS* splitting observations (b). Source-side splitting results are plotted using the same convention as Figure 4. Here we measured 7 new source-side splits across 4 different azimuths. Four of the splits, shown in green-blue, possess a similar azimuth (~150°), and produce very similar splitting parameters (Φ: 63°-69°, δt: 1.5-2.3 s). *SKS* splitting has also been conducted for Iceland (plotted as black bars) [*Xue and Allen*, 2005], with station KRO of the SIL network [*Stefánsson et al.*, 1993], nearest to our source-side measurements. The *SKS* splitting at this station is in agreement with our source-side results if we consider the azimuths of the raypaths involved. In (c), a stereographic projection of the results from (a) are plotted alongside the *SKS* result from station KRO. The distance from the centre of the circle represents the inclination of the outgoing ray from the source, and its position around the circle the azimuth. N and E stand for north and east. The event distribution for the *SKS* study is limited, with most events originating from western Pacific subduction zones with a backazimuth of ~45°. We have one source-side measurement also at a similar 45° azimuth (plotted in yellow/orange). The splitting parameters are roughly similar between the stacked *SKS* result (Φ: -63°, δt: 1.4s) and the source-side result at a similar azimuth (Φ: -36°, δt: 1.9s). This suggests that azimuthal dependency may also be present in *SKS* splitting but is harder to observe given the restricted locations of large-magnitude teleseismic earthquakes, and therefore often limited back-azimuthal sampling. The steeper inclination of *SKS* phases will also likely reduce the effect, as observed in Figure 8. In comparison to the other azimuthal patterns seen globally (Figure S7), Iceland is rather unusual in its concentric display. Such a pattern is consistent with simple transverse isotropy, or radial anisotropy, with a vertical slow axis of symmetry.

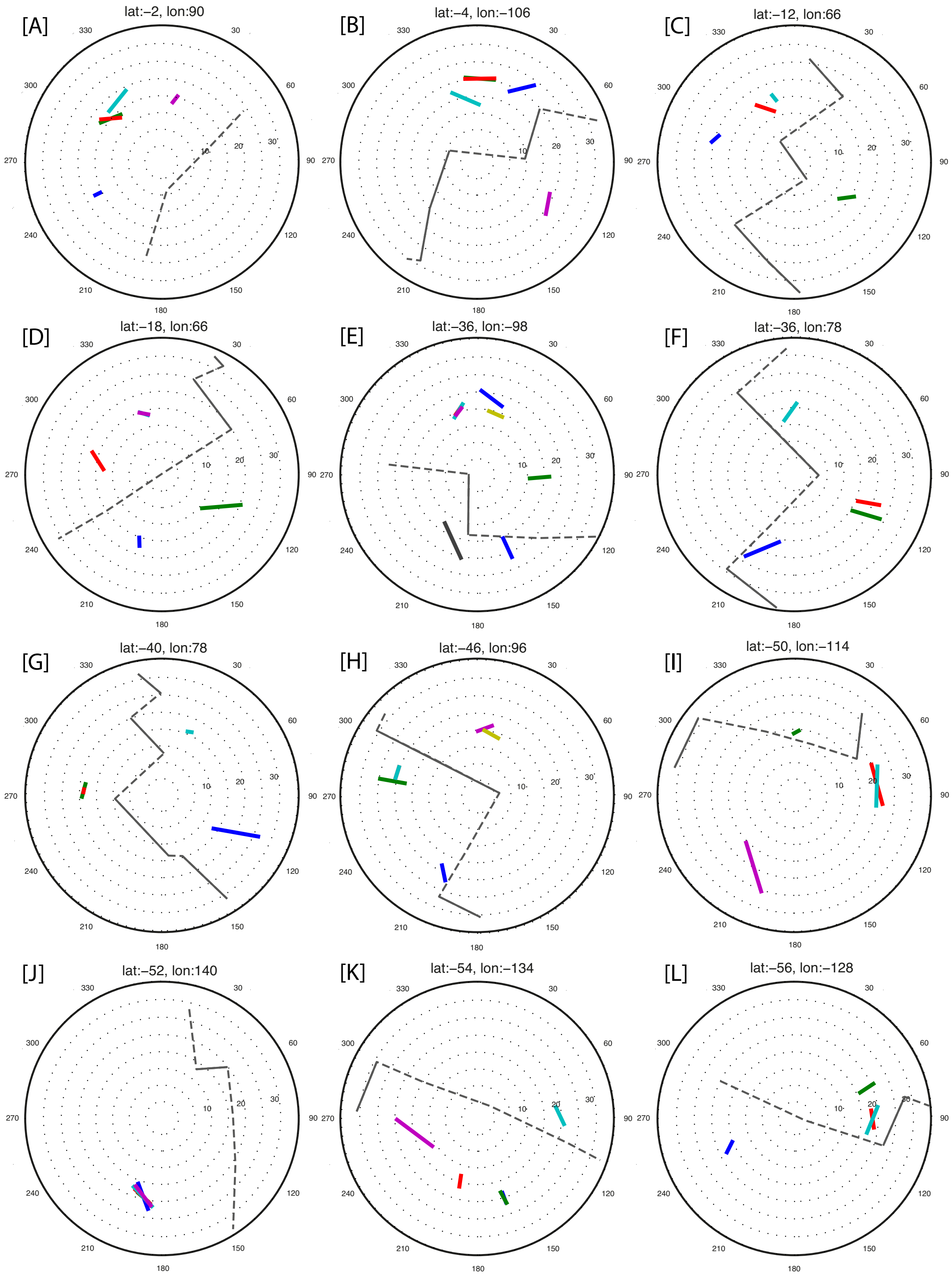
[A]
lat:–2, lon:90
[B]
lat:–4, lon:–106
[C]
lat:–12, lon:66
[D]
lat:–18, lon:66
[E]
lat:–36, lon:–98
[F]
lat:–36, lon:78
[G]
lat:–40, lon:78
[H]
lat:–46, lon:96
[I]
lat:–50, lon:–114
[J]
lat:–52, lon:140
[K]
lat:–54, lon:–134
[L]
lat:–56, lon:–128

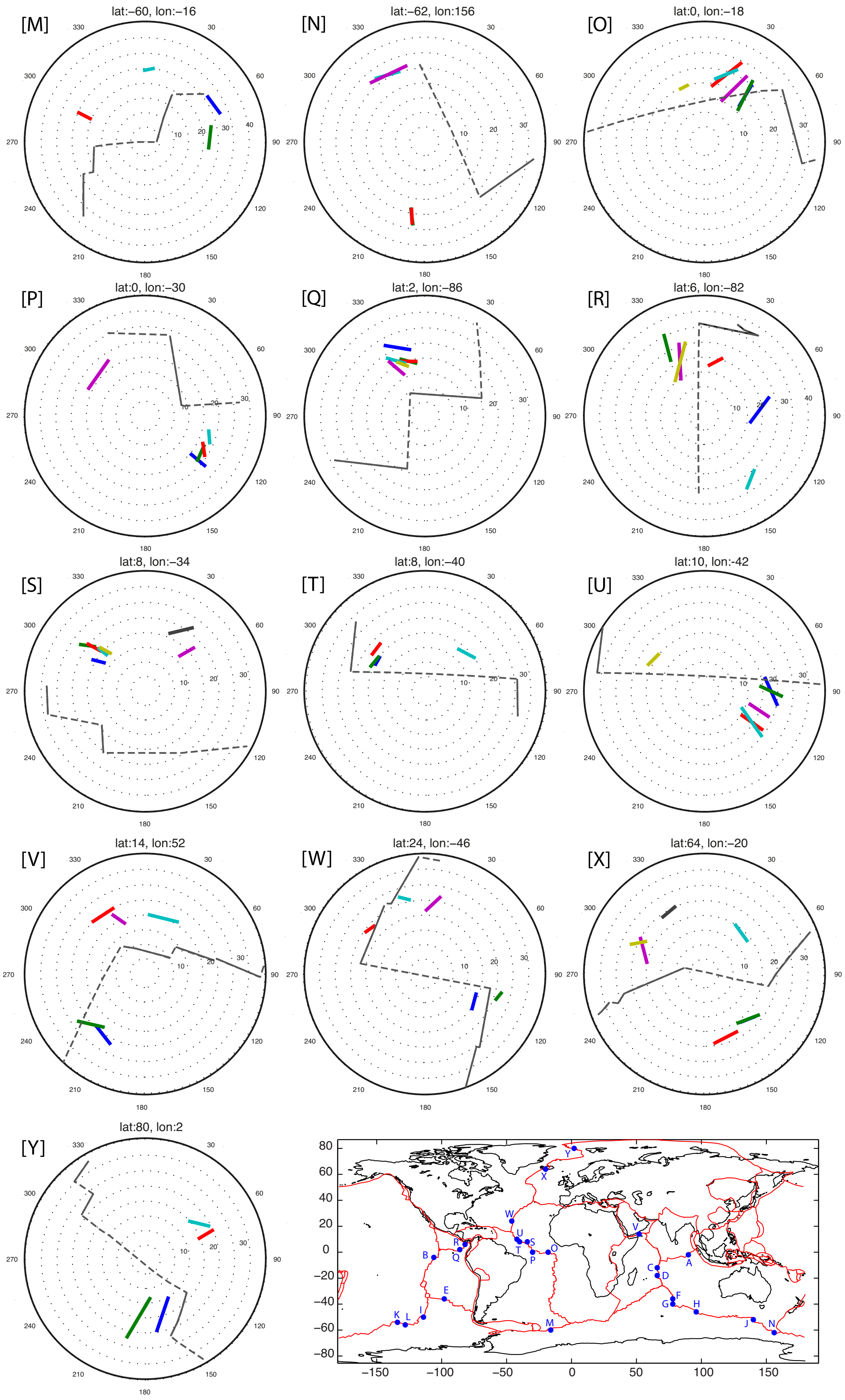

[M] lat:-60, lon:-16
[N] lat:-62, lon:156
[O] lat:0, lon:-18
[P] lat:0, lon:-30
[Q] lat:2, lon:-86
[R] lat:6, lon:-82
[S] lat:8, lon:-34
[T] lat:8, lon:-40
[U] lat:10, lon:-42
[V] lat:14, lon:52
[W] lat:24, lon:-46
[X] lat:64, lon:-20
[Y] lat:80, lon:2

Figure S7. Stereographic projections of 25 event locations with four or more source-side splitting measurements demonstrating azimuthal dependence. Individual measurements (bars) are coloured differently to give clarity (colours are unrelated to ray azimuth in this case). Measurements are plotted as a function of inclination (distance from the centre of the circle) and azimuth (angle from 0° North) of the outgoing ray from the source. Orientation of the bars represents the measured fast direction and their length is proportional to the delay time. Grey lines are a projection of the plate boundary [*Bird*, 2003] relative to the latitude-longitude coordinate of the source (labeled at the top of each stereo-plot). Dashed grey represents a transform fault and solid grey a ridge segment. The locations of each event location are plotted as blue dots on the inset map highlighting the widespread existence of azimuthal dependence. For each plot, if there is more than measurement for a given station (e.g. for an earthquake repeating in the same location), then the results will be immediately co-located, as they will possess the same ray parameter. In the majority of cases however, the multiple results plotted reflect measurements made across multiple receivers.

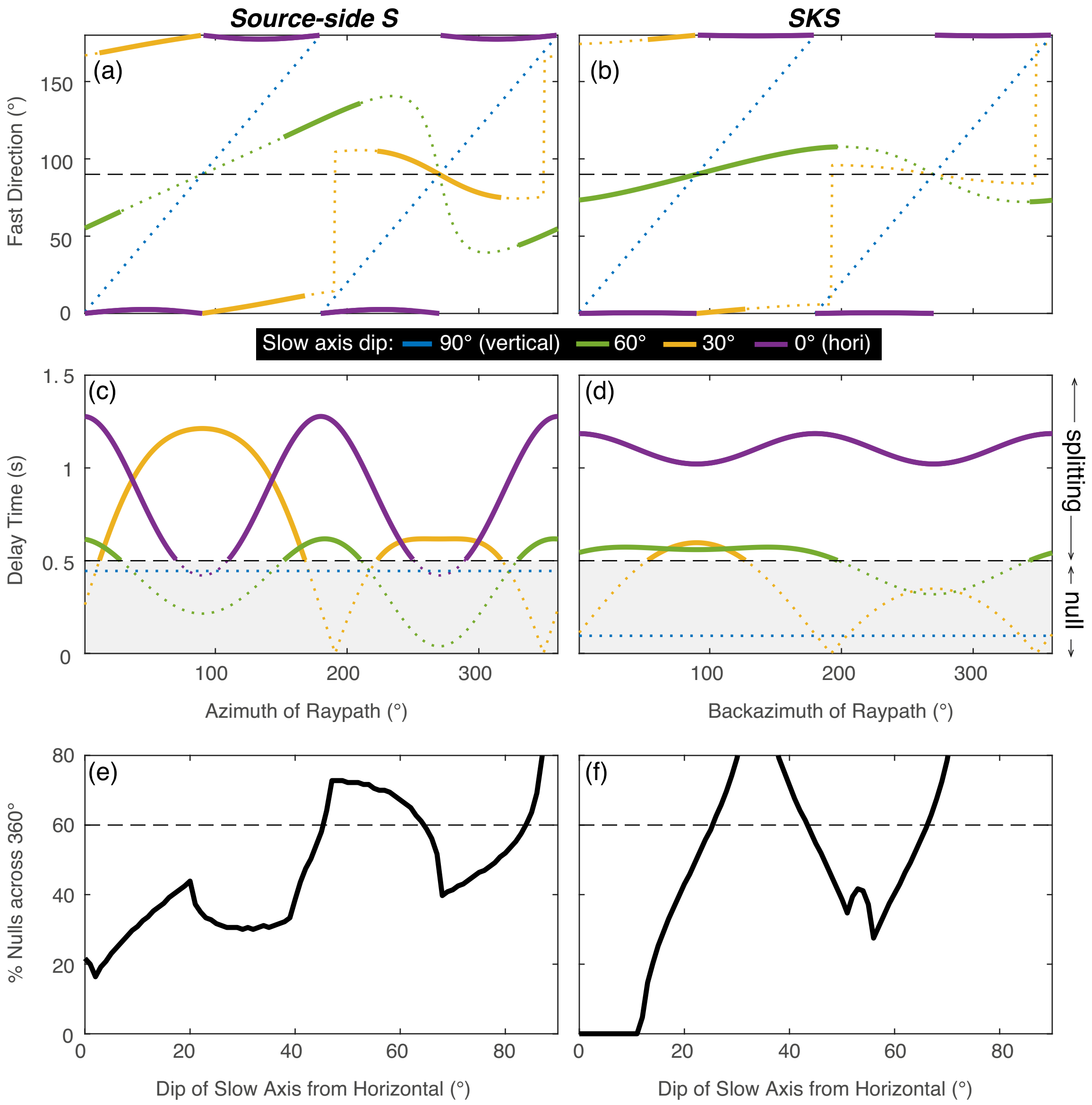

Figure S8. Same as Figure 8 but for an SPO type mechanism characterized by a slow symmetry axis, such as aligned melt (see also Figure S9 and Table S4). The melt distribution is modeled as aligned disks with an aspect ratio of 0.01 (ratio of the height of the oblate spheroid to its radius) and a melt fraction of 0.2% according to [*Tandon and Weng*, 1984]. These values were chosen for (i) consistency with [*Blackman and Kendall*, 1997] who modeled melt beneath a mid-ocean ridge, and (ii) to produce anisotropy with a maximum strength (~5%) similar to the olivine LPO model in Figure 8, following the work of [*Holtzman and Kendall*, 2010]. As the aspect ratio increases (more spherical) and/or the volume fraction of melt decreases, the strength of anisotropy would decrease, and vice-versa. Varying these melt dimension parameters would affect the predicted delay times (c-d) and percentage of nulls (e-f), but the predicted fast directions (a-b) would remain similar.

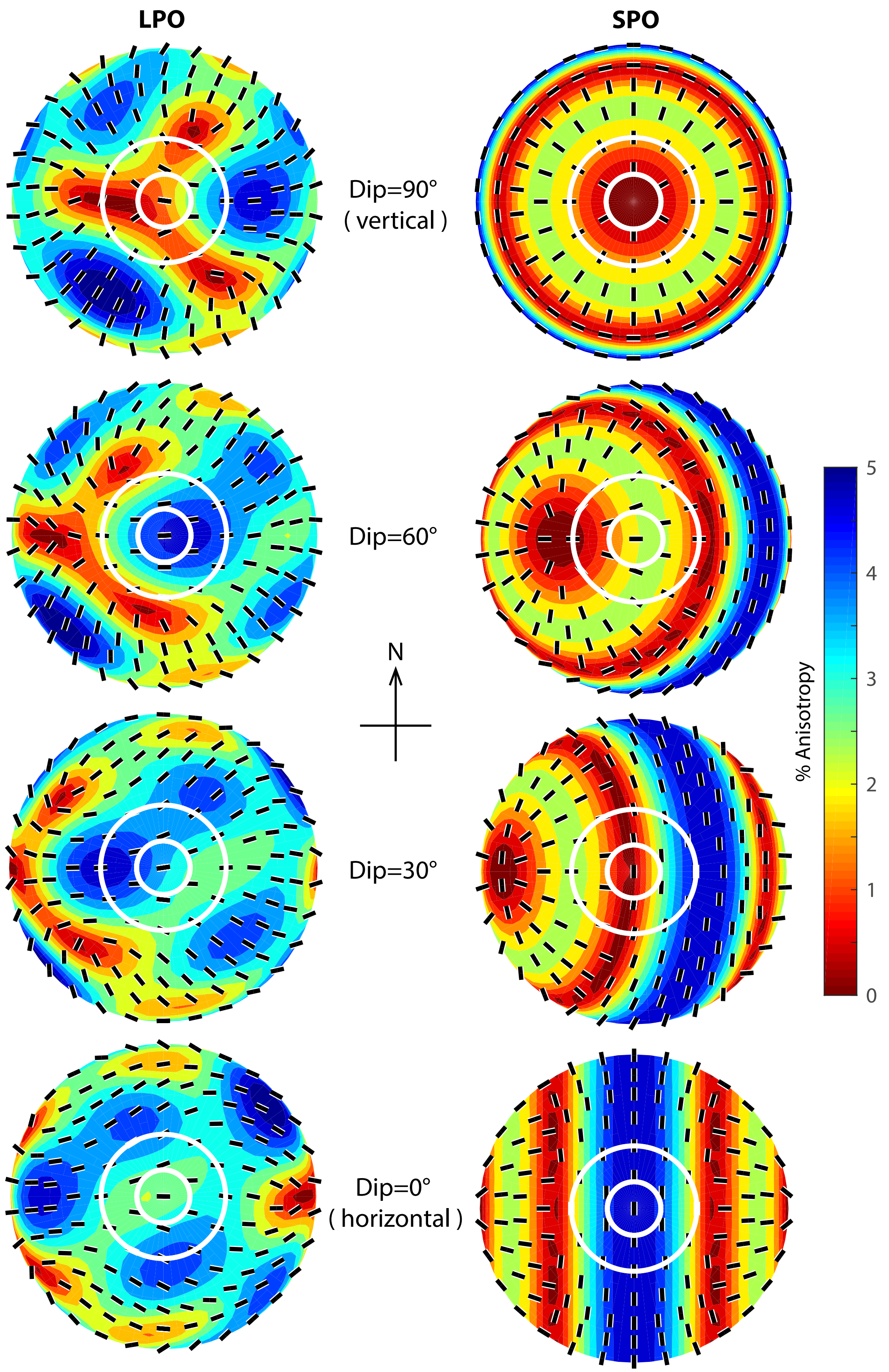

Figure S9. Shear wave polarization plots of anisotropic models and geometries used

in Figures 8 and S8. Plots were produced using the MS_plot function in MSAT [*Walker and Wookey*, 2012] and are of the lower hemisphere (looking from above). The two anisotropic models shown are; A-type olivine LPO (on the left), and SPO from aligned melt pockets (on the right). Elastic constants for each are provided in Tables S3 and S4. The dip of the symmetry axis varies from vertical to horizontal in 30° increments from top to bottom. The white circles represent 360° of azimuthal sampling for typical source-side (outer circle) and *SKS* (inner circle) raypath inclinations as plotted in Figures 8 and S8. The colours represent the strength of shear wave anisotropy according to the colour scale provided. Black bars are orientated in the fast shear wave polarization direction.

Table S1. List of "null" seismic stations used during this study for source-side splitting analysis. Station quality characteristics such as the percentage of null *SKS* results, and the time dependent station misalignment values are provided. A full description of each column is given in the third tab under "column descriptions".

Table S2. Table of results and corresponding seafloor spreading parameters used in modeling. Results are sub-divided into "nulls" and "splits" on different tabs. The third tab "Contents" gives a detailed description of each column.

236.3	84.5	81.5	0.4	3.4	0.3
84.5	218.5	82.9	-1.8	1.2	0.3
81.5	82.9	208	-1.3	6.1	0.2
0.4	-1.8	-1.3	64.9	-0.1	-1.9
3.4	1.2	6.1	-0.1	68.7	0.3
0.3	0.3	0.2	-1.9	0.3	66.6

Table S3. Elasticity tensor (C_{ij} components in GPa) for an anisotropic model of olivine A-Type LPO. Elastic constants were retrieved from page 407 of [*Karato*, 2008] and are based on experimental data of deformed olivine aggregates under typical upper mantle conditions (pressure: 5 GPa and temperature: 1573 K). Values are stated by the author to be "typical" but may vary depending on the degree of lattice-preferred orientation.

206.8	76.2	76.2	0.0	0.0	0.0
76.2	210.4	77.0	0.0	0.0	0.0
76.2	77.0	210.4	0.0	0.0	0.0
0.0	0.0	0.0	66.7	0.0	0.0
0.0	0.0	0.0	0.0	60.2	0.0
0.0	0.0	0.0	0.0	0.0	60.2

Table S4. Elasticity tensor (C_{ij} components in GPa) for SPO model of aligned melt pockets. Elastic constants were derived using the MS_effective_medium function of MSAT [*Walker and Wookey*, 2012] and effective medium theory of [*Tandon and Weng*, 1984] for unidirectional spheroid inclusions. The host matrix was defined by typical upper mantle physical properties (Vp = 8 km/s, Vs = 4.5 km/s, ρ = 3300

kg/m^3). Corresponding values for basaltic melt inclusions (Vp = 2.5 km/s, Vs = 0 km/s, ρ = 2700 kg/m^3) were taken from [*Murase et al.*, 1973; *Blackman and Kendall*, 1997]. The aspect ratio and volume fraction of the melt inclusions were chosen as 0.01 and 0.2% respectively.
